# A multi-center, randomized controlled clinical trial, cost-effectiveness and qualitative research of electroacupuncture with usual care for patients with non-acute pain after back surgery: study protocol for a randomized controlled trial

**DOI:** 10.1186/s13063-018-2461-6

**Published:** 2018-01-24

**Authors:** Byung-Cheul Shin, Jae-Heung Cho, In-Hyuk Ha, In Heo, Jun-Hwan Lee, Koh-Woon Kim, Me-riong Kim, So-Young Jung, Ojin Kwon, Nam-Kwen Kim, Haeng-Mi Son, Dong-Wuk Son, Kyung-Min Shin

**Affiliations:** 1 Spine & Joint Center, Department of Korean Rehabilitation Medicine, Pusan National University Korean Medicine Hospital, Yangsan, 50612 South Korea; 2 Division of Clinical Medicine, School of Korean Medicine, Pusan National University, Yangsan, 50612 South Korea; 3 Department of Korean Rehabilitation Medicine, Kyung Hee University, Seoul, 02447 South Korea; 4 Jaseng Spine and Joint Research Institute, Jaseng Medical Foundation, Seoul, 06017 South Korea; 5 School of Korean Medicine, Pusan National University, Yangsan, 50612 South Korea; 6 Clinical Research Division, Korea Institute of Oriental Medicine, Daejeon, 34054 South Korea; 7 Korean Medicine Life Science, Campus of Korea Institute of Oriental Medicine, University of Science & Technology (UST), Daejeon, 34054 South Korea; 8 Department of Ophthalmology & Otolaryngology and Dermatology, School of Korean Medicine, Pusan National University, Yangsan, 50612 South Korea; 9 Department of Nursing, Ulsan University, Ulsan, 44610 South Korea; 10 Department of Neurosurgery, Yangsan Pusan National University Hospital, Yangsan, 50612 South Korea

**Keywords:** Electroacupuncture, Low back pain, Back surgery, Postoperative pain, Integrative medicine, Randomized controlled trial

## Abstract

**Background:**

Although pain after back surgery is known to be difficult to control, various treatment options are available to patients and physicians. A protocol for a confirmatory randomized controlled trial (RCT) on pain and function after back surgery was designed based on the results of a pilot trial. The aim of this study is to compare the effectiveness and safety of electroacupuncture (EA) with usual care (UC) versus UC alone on pain control and functional improvement after back surgery.

**Methods/design:**

This study is a multi-center, randomized, assessor-blinded trial with an active control conducted in conjunction with a cost-effectiveness analysis and qualitative research. Participants with non-acute low back pain with or without leg pain after back surgery who have a Visual Analogue Scale (VAS) pain intensity score ≥ 50 mm will be randomly assigned to either the EA with UC group (n = 54) or the UC group (n = 54). Following randomization, participants in both groups will receive the same UC treatment twice a week for a four-week treatment period. Participants assigned to the EA with UC group will additionally receive EA twice a week for the same four-week period. The primary outcome measure will be assessed using a VAS pain intensity score for low back pain. The secondary outcomes will include the Oswestry Disability Index, EuroQol 5-Dimension score, and drug intake. The primary and secondary outcomes will be measured at one, four, and eight weeks post randomization.

**Discussion:**

The results of this study will provide evidence of the effectiveness and cost-effectiveness of EA in managing postoperative pain following back surgery. In addition, the qualitative research results will help improve the quality of integrative medical interventions.

**Trial registration:**

Clinical Research Information Service (CRIS), Republic of Korea, KCT0001939. Registered on 8 June 2016.

**Electronic supplementary material:**

The online version of this article (10.1186/s13063-018-2461-6) contains supplementary material, which is available to authorized users.

## Background

Low back pain (LBP), a widespread condition, has a high global prevalence of 10% [1]. According to the Global Burden of Disease 2010 Study, LBP is the sixth highest overall burden on the society of the 291 conditions [2] and has thus been the focus of a number of related studies [3]. Although acute LBP is usually recovered within six weeks [4], the clinical guidelines recommend surgery such as spinal fusion for chronic LBP in failure of more than two years of all other recommended conservative treatments [5]. While lumbar spine surgery methods such as discectomy and spinal fusion continue to increase in popularity, [6] various complications may occur following back surgery, possibly resulting in worse conditions [7].

The most common complication associated with back surgery is pain and approximately 40% of patients may experience pain after lumbar spine surgery [8]. Therefore, in cases in which pain persists after back surgery, pain management is essential [9, 10]. Opioid analgesic drugs are a cornerstone of postoperative pain management [11]. However, opioids may cause various side effects ranging from itching and sedation to nausea [12]; effective and safe pain management after back surgery is thus warranted.

Several clinical trials have demonstrated that acupuncture is generally safer [13, 14] and more cost-effective [15, 16] than routine care. A recent systematic review suggested that certain modes of acupuncture improved acute postoperative pain and reduced opioid use [17]. Recently, the clinical practice guidelines from the American College of Physicians in 2017 indicated that acupuncture improved pain in acute and chronic LBP [18].

Electroacupuncture (EA) is the application of electrical stimulation to acupuncture techniques. Currently, EA is a common treatment for pain management [19, 20], including myofascial pain [21], osteoarthritis of the knee [22], chronic shoulder and neck pain [23], and LBP [24]. EA is regarded to modulate pain through significant changes in bioactive chemicals such as opioids, serotonin, and norepinephrine in peripheral injury sites, the spinal cord, and supraspinal structures [20].

In our recent systematic review, we found that acupuncture analgesia for pain after back surgery yields positive results [25], but there have been few clinical trials that have reported the effectiveness of EA for pain following back surgery. Therefore, we conducted a pilot randomized controlled trial (RCT) of EA in patients with non-acute pain after back surgery between 2013 and 2014 to assess the feasibility of a full-scale study and to calculate an adequately powered sample size [26]. Using the pilot RCT results (currently being prepared for submission to a peer-reviewed journal), we calculated the sample size and tested the acceptability of the study design to assess the effectiveness and safety of EA for post-back surgery pain. We thus propose a pragmatic, confirmative, and comparative multi-center RCT design following the CONSORT [27] reporting guidelines and Standards for Reporting Interventions in Clinical Trials of Acupuncture (STRICTA) recommendations [28]. We selected usual care as the comparator in this study to reflect real-world conditions with reference to the most common treatments used in patients with LBP as assessed from the 2011 Korean Health Insurance Review and Assessment (HIRA) statistics [29] or clinical practice guidelines [30]. This study will additionally include a cost-effectiveness analysis and qualitative research.

## Methods/design

### Objective

We hypothesize that EA may have better effectiveness and cost-effectiveness with good tolerance in safety in patients with pain after back surgery when used as complementary treatment with UC. The aim of this study is to compare the effectiveness and safety of EA with UC versus UC alone on pain control and functional improvement after back surgery. Additionally, cost-effectiveness analyses will be conducted to investigate the effectiveness and/or cost-effectiveness of EA in patients with pain after back surgery; qualitative research will be conducted to understand participants’ personal experiences with symptoms, factors influencing their pain, and their perceptions of the intervention.

### Design

This study is a multi-center, randomized, assessor-blinded trial with two parallel arms and an active control. This study will be conducted in three hospitals in South Korea. Participants will be independently recruited at each hospital site through online advertisements placed on the hospital website and offline on-site advertisements, such as hospital bulletin boards, to obtain the appropriate participant enrollment and target sample size. Potential participants will be required to undergo screening to determine their eligibility. When eligible, participants will be asked to provide written informed consent, after which the baseline evaluation will be conducted by a study researcher. At screening, a clinical research coordinator (CRC) will collect information on various basic sociodemographic characteristics such as gender, age, vital signs, height, weight, and medication history. Korean medicine doctors (KMDs) will collect information on the medical history of individuals who have received spinal surgery, such as the timing of surgery, type of surgery (i.e. fusion, decompression, or discectomy), surgically involved vertebra(e), number of surgeries, time of pain, onset of symptoms, and duration of symptoms. On their second visit, screened participants who are considered eligible for trial participation based on the inclusion and exclusion criteria will be randomly allocated using a 1:1 ratio to either the EA plus UC group or the UC alone group. After randomization, the treatment procedure will be scheduled and managed by a CRC. To maximize participant retention, there will be flexibility during the visit period and researchers will allow actual visits to be within seven days of prescheduled visits. The post-treatment follow-up will occur one, four, and eight weeks following the four-week interventional period to know whether the treatments effects are maintained.

**Fig. 1:**
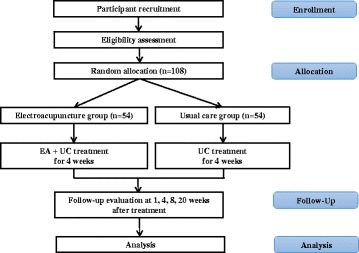
*Flow chart* showing the process of participant recruitment, treatment, and analysis

**Fig. 2:**
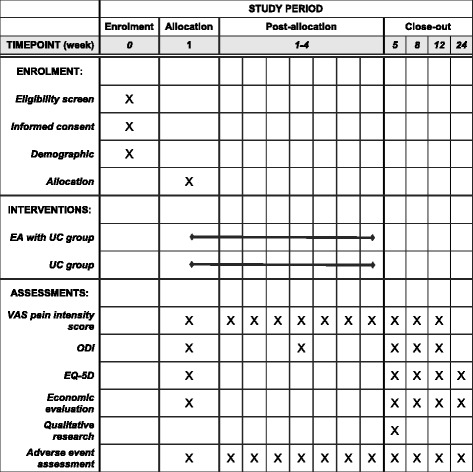
SPIRIT figure of enrolment, interventions, and assessments. EA electroacupuncture, UC usual care, VAS visual analogue scale, ODI Oswestry Disability Index, EQ-5D EuroQol five-dimensional questionnaire

All participants will be enrolled through voluntary participation and written informed consent from all participants will be obtained in accordance with the Declaration of Helsinki [31]. Trial participation may be terminated at any time during the trial through voluntary refusal to continue or in cases of significant clinical deterioration as judged by KMDs. Participants suffering from trial-related problems or adverse events (AEs) may receive medical treatment for compensation. The trial will be insured to compensate for any AEs related to the trial interventions. This study is registered at the Korea National Institutes of Health Clinical Trials Registry, “Clinical Research Information Service (https://cris.nih.go.kr/cris/en/).” It is also included in the WHO Registry Network (registry number KCT0001939). The study flow chart is presented in Fig. 1 and the trial timetable is depicted in Fig. 2.

### Participants

(Same as or similar to the pilot protocol [26].)

#### Inclusion criteria

1. Participants aged 19–70 years
2. Participants with LBP that has persisted or recurred after back surgery, regardless of leg pain, with the current pain episode continuing for three weeks or longer
3. Participants with a pain score on the VAS ≥ 50 mm
4. Participants who have voluntarily agreed to participate in the study and have provided written informed consent

#### Exclusion criteria

1. Participants diagnosed with a serious disease(s) that may potentially cause LBP (e.g. spinal infection, cancer, or inflammatory spondylitis)
2. Participants with severe neurological symptoms or progressive neurological deficits (e.g. bowel/bladder symptoms, paraplegia, or presence of neurogenic claudication)
3. Participants whose cause of pain is not the result of spine or soft tissue disease(s) (e.g. cancer, fibromyalgia, gout, or rheumatoid arthritis)
4. Participants with a chronic disease(s) that may potentially influence treatment effects or treatment result analyses (e.g. diabetic neuropathy, dementia, severe cardiovascular disease, or epilepsy)
5. Participants for whom acupuncture may potentially be unsafe or inappropriate (e.g. those with severe cardiovascular disease, hemorrhagic disease, anticoagulant therapy history, or severe diabetes at high risk of infection)
6. Participants who are currently pregnant or planning to be pregnant
7. Participants with a psychiatric disease(s) or who are currently receiving treatment for a psychiatric disease(s)
8. Participants currently participating in other clinical trial(s)

### Randomization and allocation concealment

Participants will be randomly allocated to either the EA plus UC group or the UC alone group with equal probability. Computer-generated block randomization will be used to ensure that both groups are assigned the same number of participants at a 1:1 ratio. A center-stratified block randomization scheme will be developed and administered by a statistical expert at the Korea Institute of Oriental Medicine using SAS® Version 9.4 (SAS institute Inc., Cary, NC, USA).

Sequentially numbered, sealed opaque envelopes of the same shape and size will be used to conceal group allocation and avoid selection bias at each recruitment site. Patients will be assigned to one of the two groups according to the randomization code and practitioners will deliver the allocation-appropriate treatment. Each trial participation site will store the randomization numbers in a double-locked cabinet on the hospital grounds. The allocation sequence will be concealed from the outcome assessor to prevent detection bias.

### Blinding

As the practitioners and participants in this trial cannot be blinded to the allocation of treatment groups due to the differences in the nature of the EA intervention, only the outcome assessor will be blinded. Outcome assessors will not participate in the EA or UC treatment, and they will conduct the outcome assessments in a separate room without any knowledge of participant allocation and are therefore considered safe from detection bias [32]. Unblinding the assessors will be permissible only in specific situations, such as when knowledge of the actual treatment is highly necessary for the appropriate management of participants (e.g. serious AEs (SAEs).

### Education on standardization of study procedures

First, we will develop standard operating procedures (SOPs) for the entire trial, interventions, roles, and training of assessors, researchers, and CRCs through a consensus based on experience with the previous pilot trial [26]. All researchers will be required to complete a clinical trial training in accordance with their individual roles. Licensed KMDs will be involved in this trial as practitioners or outcome assessors. Specifically, practitioners will have had three or more years of clinical experience after being certified with KMD licensure by the Korean Ministry of Health and Welfare. They will have undergone an educational course to standardize the study procedures and this process will ensure that they adhere to the study protocol and are familiar with the study interventions and their administration. All of the practitioners involved in the trial will undergo intensive training customized by role (two training sessions of a 1-h duration or more, with each session including lectures and hands-on sessions) to enable full comprehension of the EA procedure, which is semi-standardized regarding acupoints, needling depth, and manual stimulation methods. The study details, protocol, and outcome assessment process will additionally be standardized among outcome assessors through trainings based on the SOPs.

In this study, to improve the adherence to protocol, the researchers will be trained on the SOPs and regularly monitored. The participants will be thoroughly explained on the process of the study, noticed of their visit schedules via phone calls or text messages, and provided financial compensation to ensure their adherence to the protocol.

### Interventions

#### EA with UC group

**Fig. 3:**
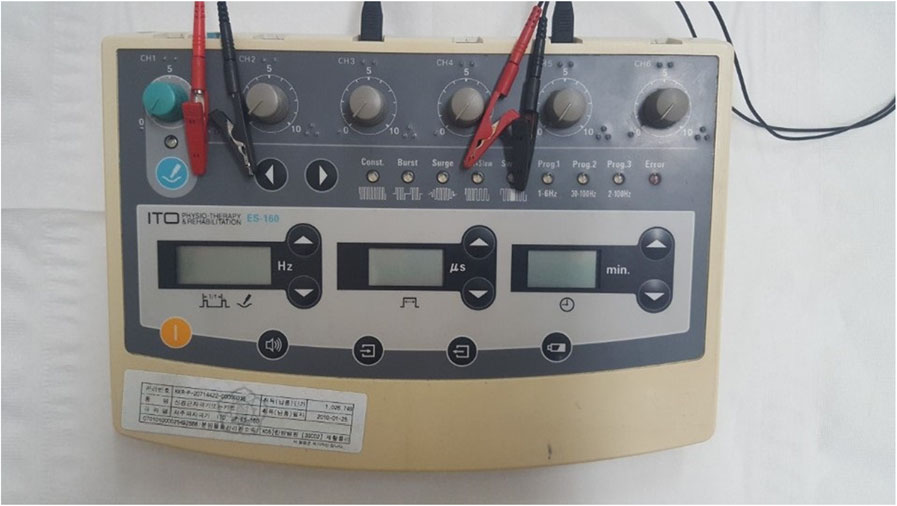
Electrical machine (ES-160, ITO Co. Ltd., Tokyo, Japan)

In the EA with UC group, both acupuncture and EA will be performed. The intervention will be administered by KMDs using 0.25-mm diameter × 40-mm length disposable stainless steel needles (Dongbang Acupuncture Inc., South Korea) and will be provided a total of eight sessions (two sessions/week) for a period of four weeks. Semi-standardized acupuncture points will be used. In other words, Jia-ji (six acupoints, bilateral Ex-B2 at L3, L4, and L5) will be used as the required and standardized points; a maximum of nine acupuncture points will be used as additional points. These additional points will be selected depending on the patients’ symptoms. Electrical stimulation will be applied to four acupuncture points of Jia-ji (bilateral Ex-B2 at L3 and L5) and performed for 15 min with a biphasic waveform current, which utilizes alternating interrupted waves and continuous waves at 50 Hz in a triangular form and a compressional wave [33] using an electronic stimulator (Fig. 3, ES-160, ITO Co. Ltd., Tokyo, Japan). The procedure for EA treatment will be designed to reflect actual clinical settings and will be based on consensus among 21 acupuncture and spinal disorder experts in several offline meetings to ensure the feasibility of the study design.

All participants will receive the same UC treatment (see below for details) for the same four-week period. Each intervention will be provided in 5- to 10-min intervals. More detailed procedures according to the STRICTA are attached in Additional file 1.

#### UC group

The UC for LBP generally consists of conventional drugs for LBP (pain medication with muscle relaxants), physical therapy, and an educational program based on LBP clinical guidelines [30]. Conventional pharmaceutical or non-pharmaceutical treatments excluding invasive interventions (e.g. analgesics and physical therapy are allowed; injections and surgery are disallowed) relevant to post-surgical LBP will be administered and monitored carefully at each visit. Physical therapy will be administered by practitioners twice a week during the treatment period (four weeks). Interferential current therapy (ICT; EF-150, OG Giken Co., Okayama, Japan; STI-300, Stratek Co. Ltd., Anyang, South Korea) and superficial heat therapy will be applied for 15 min each. These physical therapies were selected with reference to the most common treatments used in LBP patients as assessed from the 2011 Korean HIRA statistics [29] or clinical practice guidelines [30]. Participants will receive a standardized educational program on LBP, comprising a 20-min video and a brochure, which includes information on the post-surgical pain prevalence, pathophysiology, and management tips for preventing LBP while performing everyday activities through exercise, such as cat and camel, single knee to chest, and bridging exercise.

This confirmatory, multi-center, pragmatic RCT is designed to investigate the effectiveness, as opposed to the efficacy, of EA combined with UC compared to UC alone in patients with non-acute pain following back surgery. Therefore, neither a placebo nor sham EA will be employed as an active control [34]; additionally, this trial aims to assess the effectiveness, safety, and cost-effectiveness under settings reflecting real-world conditions.

### Outcome measurements

#### Primary outcome

The primary outcome of this study is the pain intensity of LBP after back surgery as assessed by a VAS. Post-surgical LBP severity will be quantified using a VAS pain intensity score as determined on a 100-mm horizontal line, where 0 indicates no pain and 100 indicates intolerable pain [35, 36]. The level of back pain during the previous week will be assessed using a 100-mm pain VAS at baseline (visit 2), before each treatment session (visits 2–9), and at each post-treatment follow-up visit (visits 10–12); the primary endpoint will be VAS pain intensity score at visit 10, representing treatment completion. The outcome measures will be assessed before treatment at each visit to ascertain the effectiveness of the previous treatment(s).

#### Secondary outcome

Regarding secondary outcomes, the Oswestry Disability Index (ODI) will be used to assess disability associated with back pain [37] and the EuroQol 5-Dimension (EQ-5D) scale [38] will be employed for health-related quality of life.

The ODI consists of ten questions pertaining to daily activities and covers the following: experiencing general pain, practicing self-care (e.g. washing, dressing), lifting objects, sitting, standing, walking, sleeping, travelling, engaging in sexual activity if applicable, and participating in social activities. The items are rated on 6-point scales scored in the range of 0–5, with higher scores indicating higher pain-associated disability. The participants will be asked to complete the validated Korean version [39] of the ODI before treatment on the second and sixth visits and at each post-treatment follow-up (visits 10–12).

The validated Korean version of the EQ-5D [40] will be used to assess health-related quality of life in post-surgical LBP patients. The EQ-5D consists of five dimensions: usual activities; mobility; pain/discomfort; anxiety/depression; and self-care. The scales for these dimensions are in the range of 1–3 and lower scores represent a better health status. The EQ-5D scores will be evaluated before treatment at the initial treatment session (second visit) and at each follow-up (visits 10–13). For cost-effectiveness analysis, EQ-5D will be additionally evaluated via telephone at the 13th visit.

Changes in medicine intake and dosage will be investigated through interviews at each visit. Additional pain-related treatments will be allowed and the type and frequency will be recorded as part of the feasibility design (e.g. physiotherapy, medication).

### Adverse events

Any AEs will be monitored and reported by the researchers at each visit. All expected and unexpected AEs potentially related to the study will be monitored and their progress will be recorded until resolved. The physicians will decide whether trial participation should be discontinued based on these reports.

### Sample size

An appropriately powered full-scale sample size has been estimated using the mean between-group difference in the primary endpoint (VAS pain intensity score for LBP) identified in the previous pilot study. Based on intention-to-treat (ITT) analysis, the mean difference and standard deviation (SD) in the VAS pain intensity score for LBP between the EA plus UC group and UC alone group is estimated to be 14.02 mm ± 22.12 mm. Although this reduction in pain intensity is not moderately clinically meaningful, it does represent a minimally important difference [41].

The total number of individuals required per group is 40, considering a two-tailed test with 80% test power and a 5% significance level. After accounting for a 25% drop-out rate and the 1:1 allocation ratio, the final sample size per group has been calculated to be 54, resulting in a total sample size estimate of 108 for a full power analysis.

### Statistical analysis

The statistical analysis will be conducted using both the ITT and per-protocol (PP) analysis principles in parallel. Multiple imputation methods will be used for missing data in the ITT analysis. A PP analysis will be performed as a secondary analysis excluding data from drop-outs for any reason. An interim analysis will not be performed. An independent t-test or Wilcoxon rank sum test will be used to analyze continuous data to test for significance in between-group differences and will be presented as the mean ± SD. Chi-square test or Fisher’s exact test will be conducted for categorical data and the data will be described as the frequency and percentage (%). To analyze the VAS pain intensity score as the primary outcome, we will perform an analysis of covariance (ANCOVA) considering the baseline scores as covariates and treatment group as the fixed factor. A paired t-test or Wilcoxon signed rank test will be performed to analyze within-group differences pre and post treatment. Repeated measure analysis of variance (RMANOVA) will be used to analyze the between-group differences at each visit. Subgroup analysis will be conducted according to age, gender, and medical history (e.g. the existence of osteoporosis, pre-surgery diagnoses), which may have the possibility of affecting the treatment effects on each outcome.

A safety assessment will be performed for all AEs occurring during the study period. The incidence of AEs, AEs leading to withdrawal, and SAEs will be summarized by group and analyzed using Fisher’s exact test or chi-square test.

All statistical analyses will be conducted by a designated statistician blinded to group allocation with SAS® Version 9.4 (SAS institute Inc., Cary, NC, USA) for Windows. The level of significance will be set at 5%.

### Cost-effectiveness analysis

The economic evaluation study will be conducted along with the comparative effectiveness RCT. We aim to estimate the short-term cost-effectiveness of EA with UC compared to UC alone from the South Korean societal perspective. Cost components of both treatments will be defined and measured according to the identification, measurement, and valuation process. Cost data from all individual participants will be obtained using separately developed economic case report forms. Effectiveness and utility measurements will be collected from the main comparative effectiveness RCT. Quality of life will be measured by the EQ-5D instrument; quality-adjusted life years (QALYs) gained in both groups will be calculated using the area under the curve method. Cost and effectiveness (utility) will be analyzed as intention to treat and missing data will be imputed using multiple imputations. In the deterministic analysis, mean values of cost and effectiveness (utility), derived from generalized linear model adjusting for potential confounders, will be used as a representative value for calculating ICER (incremental cost-effectiveness ratio). We will use the bootstrapping percentile method to identify the sampling uncertainty of ICER. Cost-effective planes and cost-effectiveness acceptability curves will also be estimated to display and interpret statistical uncertainty and economic decision-making according to the maximum willingness to pay. If we could not observe the fully yield cost and effective outcomes of both alternatives in this short-term within trial evaluation, we will develop a decision analytic model and conduct a longer-term period evaluation.

### Qualitative research

The qualitative research will be conducted with participants receiving EA and UC. We aim to describe the meaning of these participants’ personal experiences, perceptions, and expectations of EA with UC. Twenty participants receiving eight sessions of EA plus UC, consisting of EA, physical therapy, pain medication, and patient education two times/week at the three Korean medicine hospital sites, will be included in the qualitative analysis. Qualitative interviews will be conducted at four weeks after the completion of treatment (10th visit) by trained researchers. Additional informed consent for the qualitative research will be obtained and incentives will be given to participants as an ethical consideration. The data will be collected through in-depth individual interviews with audio recordings and verbatim transcriptions. All of the text will be read line-by-line for coding and the data will be processed using qualitative content analysis. The codes will be classified according to concepts, categories and subcategories by abstractness. The data will be compared for interrater reliability with regard to the researchers’ theoretical sensitivity. To ensure the rigor of the study, the researchers will consider the credibility, fittingness, auditability, and confirmability. The credibility of the data will be enhanced by using in-depth interviews that reflected the participants’ own experiences. Theoretical saturation of the data and the researchers’ theoretical sensitivity will be added fittingness will be enhanced by providing demographic and illness-related data of the participants along with various incidents and events in the texts. Auditability will be ensured by providing analysis procedures in detail and by showing conclusions explicitly linked with displayed data. Confirmability, which refers to a freedom from possible research biases, will be achieved by undertaking the strategies discussed above.

### Data collection, management, and monitoring

The data will be collected through paper-based documents written by the CRC and outcome assessors and will then be entered into web-based case report forms on electronic data capture (EDC) systems. The entry and coding of clinical data and the data management and reporting will be conducted using the Medidata RAVE data management system (Medidata Solutions, Inc., New York, NY, USA). Data management will be performed in compliance with the trial’s SOPs. AEs will be categorized following the Medical Dictionary for Regulatory Activities, MedDRA Ver. 19.0 (MedDRA Maintenance and Support Services Organization, VA, USA). All original data sources including consent forms, questionnaires, medical history, and other relevant records will be stored at each study site in limited-access areas for three years.

This study will be monitored by the Korea Institute of Oriental Medicine. During the study period, the clinical research associate will monitor the written informed consent documents, recruitment status, protocol compliance, overall trial progress, data quality, timeliness of data collection, treatment administration, and other relevant trial aspects and processes. The Ministry of Food & Drug Safety (MFDS) in Korea will carry out audits at regular intervals.

## Discussion

EA is a common treatment for pain management and has shown a greater analgesic effect than placebo; reductions in non-specific LBP scores using a 100-point scale were > 20 points greater in EA than in placebo [42]. EA is currently widely used to treat postoperative pain [43, 44]. However, after summarizing the latest evidence in a 2015 systematic review on the effects of acupuncture or EA on treating acute post-back surgery pain, the conclusions were promising but not confirmative [25]

Moreover, there is a paucity of clinical trials investigating the effectiveness of EA for managing non-acute postoperative pain following back surgery. The authors therefore conducted a pilot RCT to assess the feasibility of a full-scale clinical trial [26]. Based on the pilot RCT results, the authors report the protocol design for a multi-center, confirmatory, pragmatic RCT with an additional cost-effectiveness and qualitative research. The study results will determine the effectiveness and safety of using EA combined with UC compared to UC alone in managing non-acute postoperative pain following back surgery.

Some strengths of this study include its design, as it represents a feasible, pragmatic, effectiveness trial that reflects real-world clinical conditions. In addition, the reports on this trial protocol adhere to the CONSORT statements [27] and STRICTA [28]. The Standard Protocol Items:Recommendations for Interventional Trials (SPIRIT) checklist for this protocol is attached in Additional file 2.

This protocol aims to provide evidence of the effectiveness and cost-effectiveness of EA in managing postoperative pain following back surgery. In addition, the qualitative research results will help improve the quality of integrative medical interventions by incorporating patient preferences in selecting medical services and determining health behaviors.

### Study limitations

In this study, stratified randomization by age, gender, and medical history (e.g. the existence of osteoporosis, pre-surgery diagnoses) will not be performed. Therefore, not all potential confounding factors may have the possibility of not being controlled between groups even though subgroup analysis will be conducted to know the influence of confounding factors. Also, due to the trial design to investigate effectiveness of EA, neither a placebo nor sham acupuncture (or EA) will not be used as control, so practitioner and participant blinding are not possible.

### Trial status

Recruitment began in June 2016.

## Additional files

Additional file 1: Standards for Reporting Interventions in Clinical Trials of Acupuncture (STRICTA). (DOCX 24 kb)

Additional file 2: SPIRIT 2013 checklist. (DOC 137 kb)

## Abbreviations

AE: Adverse events
CRC: Clinical research coordinator
EA: Electroacupuncture
EQ-5D: EuroQol 5-Dimension
IRB: Institutional review board
ITT: Intention-to-treat
KMD: Korean medical doctor
LBP: Low back pain
ODI: Oswestry Disability Index
PP: Per-protocol
QALYs: Quality-adjusted life years
RCT: Randomized controlled trial
SOP: Standard operating procedure
UC: Usual care
VAS: Visual analogue scale
