# Supplementary material for: A multi-center, randomized controlled clinical trial, cost-effectiveness and qualitative research of electroacupuncture with usual care for patients with non-acute pain after back surgery: study protocol for a randomized controlled trial
Source: Trials. 2018 Jan 24;19:65. doi: 10.1186/s13063-018-2461-6 (PMC5784658; doi:10.1186/s13063-018-2461-6)
Supplement: Supplementary file 1 — Standards for Reporting Interventions in Clinical Trials of Acupuncture (STRICTA). (DOCX 24 kb) [file 13063_2018_2461_MOESM1_ESM.docx]

**Appendix 1. Acupuncture treatment details as recommended by STRICTA**

|  | Item | Details |
| --- | --- | --- |
| 1. Acupuncture rationale | (a) Style of acupuncture | Traditional Korean medical theory |
|  | (b) Reasoning for treatment provided, based on historical context, literature sources, and consensus methods, with references where appropriate | -Textbook on acupuncture and moxibustion  -related articles and references[1-5]  -consensus of 21 acupuncture and spinal disorder experts at several offline meetings |
|  | (c) Extent to which treatment was varied | Partially individualised acupuncture treatment, i.e., fixed points plus additional points based on symptoms |
| 2. Details of needling | (a) Number of needle insertions per subject per session (mean and range where relevant) | From 6 to 15 |
|  | (b) Names (or location if no standard name) of points used (uni/bilateral) | - Six fixed points: Ex-B2 (0.5 cun lateral to the lower border of L3-L5 spinous process [6]; bilateral)  - additional points based on individual symptoms (0-9 points) |
|  | (c) Depth of insertion, based on a specified unit of measurement, or on a particular tissue level | 10 to 20 mm |
|  | (d) Response sought (eg, de qi or muscle twitch response) | 'De qi' sensation and muscle contraction |
|  | (e) Needle stimulation (eg, manual, electrical) | Electrical biphasic waveform current to four fixed points of Ex-B2 (L3 and L5; bilateral)  (Compressional wave that combines an interrupted wave and a continuous wave, in triangular form, at a frequency of 50 Hz by electronic stimulator (ES-160, ITO Co. Ltd., Tokyo, Japan)) |
|  | (f) Needle retention time | 15 min |
|  | (g) Needle type (diameter, length, and manufacturer or material) | A sterilised stainless steel needle (0.25 mm diameter × 40 mm length, Dongbang Acupuncture Inc., Korea) |
| 3. Treatment regimen | (a) Number of treatment sessions | Eight |
|  | (b) Frequency and duration of treatment sessions | 2 sessions/week for four weeks |
| 4. Other components of treatment | (a) Details of other interventions administered to the acupuncture group (eg, moxibustion, cupping, herbs, exercises, lifestyle advice) | - |
|  | (b) Setting and context of treatment, including instructions to practitioners, and information and explanations to patients | - |
| 5. Practitioner background | Description of participating acupuncturists (qualification or professional affiliation, years in acupuncture practice, other relevant experience) | Korean Medicine Doctor (KMD) who have had 3 or more years of clinical experience after being certified with KMD licensure by the Korean Ministry of Health and Welfare. |
| 6. Control or comparator interventions | (a) Rationale for the control or comparator in the context of the research question, with sources that justify this choice | 2011 Korean Health Insurance Review and Assessment (HIRA) statistics[7] or clinical practice guidelines[8] |
|  | (b) Precise description of the control or comparator. If sham acupuncture or any other type of acupuncture-like control is used, provide details as for Items 1 to 3 above. | - Both group receive usual care, which includes physical therapy (Interferential current therapy and superficial heat therapy), and an educational program based on LBP clinical guidelines.  - Frequency and duration (physiotherapy): 15 min / 1 session, 2 sessions a week for four weeks  - Conventional pharmaceutical or non-pharmaceutical treatments excluding invasive interventions (e.g., analgesics and physical therapy are allowed; injections and surgery are disallowed) relevant to post-surgical LBP will be allowed. |

1. Committee on Compilation of Textbook in Society for Acupuncture & Moxibustion: **Acupuncture and Moxibustion Medicine**. Paju: Jipmoondang Publishing Company; 2012.

2. Heo J, Ahn S, Kim N, Jeong C, Cha W: **Donguibogam**. Seoul: Ministry of Health & Welfare; 2012.

3. Yuan J, Kerr D, Park J, Liu X, McDonough S: **Treatment regimens of acupuncture for low back pain—a systematic review**. *Complementary Therapies in Medicine* 2008, **16**(5):295-304.

4. Xin Z-p, Cao H, He Y-h, Qiu M-l, Zheng X, Sao P: **Observation of therapeutic effect of electroacupuncture jiaji therapy for failed back surgery syndrome**. *Liaoning J Trad Chin Med* 2009, **36**:813-814.

5. Sun Z-r, Yue J-h, Zhang Q-h: **Electroacupuncture at Jing-jiaji points for neck pain caused by cervical spondylosis: a study protocol for a randomized controlled pilot trial**. *Trials* 2013, **14**(1):360.

6. Cabioglu MT, Arslan G: **Neurophysiologic basis of back-Shu and Huatuo-Jiaji points**. *The American journal of Chinese medicine* 2008, **36**(03):473-479.

7. Ahn Y-J, Shin J-S, Lee J, Lee YJ, Kim M-R, Park KB, Lee J-H, Shin K-M, Ha I-H: **Evaluation of use and cost of medical care of common lumbar disorders in Korea: cross-sectional study of Korean Health Insurance Review and Assessment Service National Patient Sample data**. *BMJ open* 2016, **6**(9):e012432.

8. **Guideline for the evidence-informed primary care management of low back pain** [<https://www.guideline.gov/summaries/summary/37954/guideline-for-the-evidenceinformed-primary-care-management-of-low-back-pain?q=low+back+pain>]
